# Energizing collaborative industry-academia learning: a present case and future visions

**DOI:** 10.1186/s40309-022-00196-5

**Published:** 2022-04-25

**Authors:** Petri Kettunen, Janne Järvinen, Tommi Mikkonen, Tomi Männistö

**Affiliations:** 1 Department of Computer Science, University of Helsinki, Helsinki, Finland; 2 Business Finland, Helsinki, Finland; 3 Faculty of Information Technology, University of Jyväskylä, Jyväskylä, Finland

**Keywords:** Industry-Academia Collaboration, Learning networks, Innovation ecosystems

## Abstract

In Industry-Academia Collaborations (IAC) both academic, scientific research results and industrial practitioner findings and experiences are produced. Both types of knowledge should be gathered, codified, and disseminated efficiently and effectively. This paper investigates a recent (2014–2017) large-scale IAC R&D&I program case (Need for Speed, N4S) from a learning perspective. It was one of the programs in the Finnish SHOK (Strategic Centres of Science, Technology, and Innovation) system. The theoretical bases are in innovation management, knowledge management, and higher education (university) pedagogy. In the future, IAC projects should be more and more commonplace since major innovations are hardly ever done in isolation, not even by the largest companies. Both intra-organizational and inter-organizational learning networks are increasingly critical success factors. Collaborative learning capabilities will thus be required more often from all the participating parties. Efficient and effective knowledge creation and sharing are underpinning future core competencies. In this paper, we present and evaluate a collaboratively created and publicly shared digital knowledge repository called “Treasure Chest” produced during our case program. The starting point was a jointly created Strategic Research and Innovation Agenda (SRIA), which defined the main research themes and listed motivating research questions to begin with—i.e., intended learning outcomes (ILO). During the 4-year program, our collaborative industry-academia (I-A) learning process produced a range of theoretical and empirical results, which were iteratively collected and packaged into the Treasure Chest repository. Outstandingly, it contained, in addition to traditional research documents, narratives of the industrial learning experiences and more than 100 actionable knowledge items. In conclusion, our vision of the future is that such transparently shared, ambitious, and versatile outcome goals with a continuous integrative collection of the results are keys to effective networked I-A collaboration and learning. In that way, the N4S largely avoided the general problem of often conflicting motives between industrial firms seeking answers and applied solutions to their immediate practical problems and academic researchers aiming at more generalizable knowledge creation and high-quality scientific publications.

## Introduction

In Industry-Academia Collaborations (IAC), both academic, scientific research results and industrial practitioner findings and experiences are produced. Both types of knowledge should be gathered, codified, and disseminated efficiently and effectively.

This paper investigates a recent large-scale IAC R&D&I program case called Need for Speed (N4S) [1]. The industry-driven research program was executed in 2014–2017. It was at that time the biggest Finnish national investment in software-related research with a budget of over 50 M€ involving 40 leading Finnish software-intensive companies and research organizations. In total, roughly 500 people participated in the program over the years.

We investigate the N4S IAC from knowledge creation and learning perspectives. The theoretical bases are in innovation management, knowledge management, and higher education (university) pedagogy.

During the 4-year program, our energized, collaborative I-A learning process produced a wide range of theoretical and empirical N4S consortia results, which were iteratively collected and jointly packaged into the shared repository called Treasure Chest available in the public domain. It helps companies to make use of the possibilities of digitalization and provides also advices for post-digitalization activities. The authors participated in the program. The second and third authors led the program representing the industrial and academic perspectives, respectively.

The rest of this paper is organized as follows. The following section frames the empirical landscape of industry-academia collaboration with recognized success factors and challenges. The next section describes our N4S case, and the succeeding section presents the empirical results. We then discuss the findings, experiences, and lessons learned with managerial and theoretical implications. Finally, we conclude with practical suggestions and pointers to further research work.

### Challenges and success factors of effective industry-academia collaboration

In the future, IAC projects will probably be more and more commonplace since major innovations are hardly ever done in isolation, not even by the largest companies. Especially the current and future grand challenges of for example energy systems transformations coupled with digitalization require multidisciplinary research and new knowledge creation and acquisition in many different domains. Often, no single company possesses all. There are increasing needs for bi-directional knowledge co-creation and technology transfers between industry and academia.

Software is increasingly a key enabling technology (KET) for industrial innovations also in non-ICT companies. Since the pace of product development is accelerating in almost all industry sectors, companies need speed for their software creation and production processes.

In academic context, empirical software engineering research has been advancing for decades. However, in order to produce practical value and utility, the research knowledge and technological development must be transferred to industrial companies in actionable forms.

There are also increasing demands for transferring knowledge and new technology the other way around from software-related industries to academia in order to inform researchers about relevant research questions, industrial opportunities, and practitioners’ challenges. To be effective, such knowledge and technology transfer requires often industrial domain knowledge and practical experience not necessarily possessed by academic software researchers.

Overall, it follows that there are increasing needs and demands for effective IAC research endeavors. However, like highlighted above, there are many challenges to overcome. On the other hand, a lot is known about the key success factors of IAC programs.

IAC has been investigated quite extensively over the years in many different disciplines and from multiple viewpoints (e.g., [2–4]). It has also been examined in the context of software research (e.g., [5–9]).

Table 1 presents an aggregated summary of the literature review on typical challenges and success factors of effective IAC in the software research domain. Notably, there are already prior publications describing and evaluating the N4S program’s overall research and development approach [10, 11]. Those are included in Table 1.

**Table 1:** Prior and related works on IAC

Themes	Results, experiences, and suggestions
Success factors	• Lean Research Approach: business cases (defined by industrial organizations with business impact); agile continuous planning and research sprints; transparent information, artifact, and asset sharing [10]
	• Research sprints (3 months) for continuous, direct business impact, 1-n and n-1 relations between research and industrial organizations (scaling), fast pace and rhythm of joint interaction occasions (program-wide quarterly review meetings), considering also non-technical changes and impacts in the particular industrial contexts, mindset and attitude towards co-creation, company co-operation, and benchmarking supported by researchers; academic researchers genuinely understanding and even anticipating specific industrial needs and technological developments [11]
	• Buy-in and support from company management, champion at the company [12]
	• Need orientation (addressing perceived real-life industry problems and possibilities), management engagement (problem formulation and research conduct); Collaborative research should be agile [13].
	• Close collaboration realized with applied agile methodologies (Scrum): 6-month sprints, collaboration ceremonies (monthly stands and retrospectives); collaboration at different levels between companies and universities with frequent opportunities to meet [14]
	• Working as one team, identifying the “right” (SE research) problem, ensuring practicality and applicability, conducting cost-benefit analysis, maturity of research prototype tools, encouraging further adoptions [8]
	• Sustainable long-term research collaboration with mutual trust and respect coming with working and spending time together; industry management commitment, champion as the main driver of the collaboration on the industry-side; researchers’ social skills; awareness of the industrial expectations and commitment to deliver accordingly; Tying the research into the daily work at the industry partner; Understanding how the qualitative information could be combined with the quantitative data in the industrial context [15]
	• Selecting an appropriate research methodology based on the specific primary research objectives and the scope of the research [9]
	• Design science approach: Producing viable artifacts that companies appreciate, research activities easily integrated into the company daily business and day-to-day work of practitioners; Industry champion driving the collaboration from the industry side, joint team based on a mutual learning and exchange of knowledge [16]
Difficulties and problems	• Funding organizations expecting linear up-front research proposals and plans (waterfallish) [10]
	• Company strategy and technology changes, collaborative and iterative way of working not suiting everybody [10]
	• Academics learning to be agile toward industry needs, practitioners learning to appreciate research rigor requires time and continuous reflection efforts; Industry and academia having different objectives and incentives [13].
	• Academia and industry having by nature different governing variables, goals, and pacing; working jointly during the period of understanding the problem, organizing and executing the joint work, communicating with different stakeholders; scaling I-A research [14]
	• Knowledge exchange vs. technology transfer; industrial challenge vs. actual problem; Industry deadlines and budgets overriding; systemic problems in the academic system (academic reward system); earning mutual trust and respect [15]
	• Mismatch between practitioners and researchers expectations [16] • Industrial companies having limited resources (especially time) for academic research related “extra work”; making research organizations to work jointly rather than even competing with each other [11]

In general, both intra-organizational and inter-organizational learning networks are increasingly considered critical success factors. Collaborative learning capabilities will thus be required more often from all the participating parties. Efficient and effective knowledge creation and sharing are then underpinning future core competencies.

### Case Need for Speed (N4S)

The Need for Speed (N4S) research program was funded by Tekes (nowadays Business Finland) as the Finnish SHOK (Strategic Centres of Science, Technology, and Innovation) program in 2014–2017 [17]. The consortia consisted initially of 11 large industrial organizations, 14 SMEs, and 10 research institutes and universities.

All the authors of the present paper participated in the program. The second author acted as the program leader (Focus Area Director, FAD) and the third author as the academic coordinator (ACO). The second actor was at the time employed by the so-called driver company of the program. Moreover, the first author contributed especially to the Treasure Chest development and dissemination.

The overarching ambition of the N4S program was stated as follows: “N4S will create the foundation for the Finnish software intensive businesses in the new digital economy”. Consequently, the long-term plan of N4S was to serve other companies where software plays a dominant role—by making the program’s results, tools, and processes widely available.

The starting point of the N4S program was a jointly created Strategic Research and Innovation Agenda (SRIA) [18]. It defined the strategic main research themes and listed motivating research questions to begin with as follows:

“N4S adopts a real-time experimental business model and provides capability for instant value delivery based upon deep customer insight”:

1. Delivering value in real time
2. Deep customer insight—better business hit-rate
3. Mercury business—find the new money

In the SRIA document, each of the three above research themes (breakthrough targets) was further elaborated with specific focus areas, goals, and envisioned results. There were motivating and engaging metaphors like “Goal-Driven Hunting Culture” for the Mercury Business and instant value delivery by just “pushing one button”.

In addition to the strategic research goals stated in the SRIA, each industrial partner company defined at least one business case [19]. There were 49 cases defined in the beginning of the program. Each case had an industrial business owner and an academic research coordinator. Typically multiple research partners worked on each business case.

## Results

In this paper, we contribute by exhibiting and analyzing the collaboratively created and publicly shared digital knowledge repository called Treasure Chest produced during the N4S program. Conceptually, the Treasure Chest comprises the following main elements:

1. Knowledge items
2. Viewing, filtering, and searching mechanisms for accessing them.

**Fig. 1:**
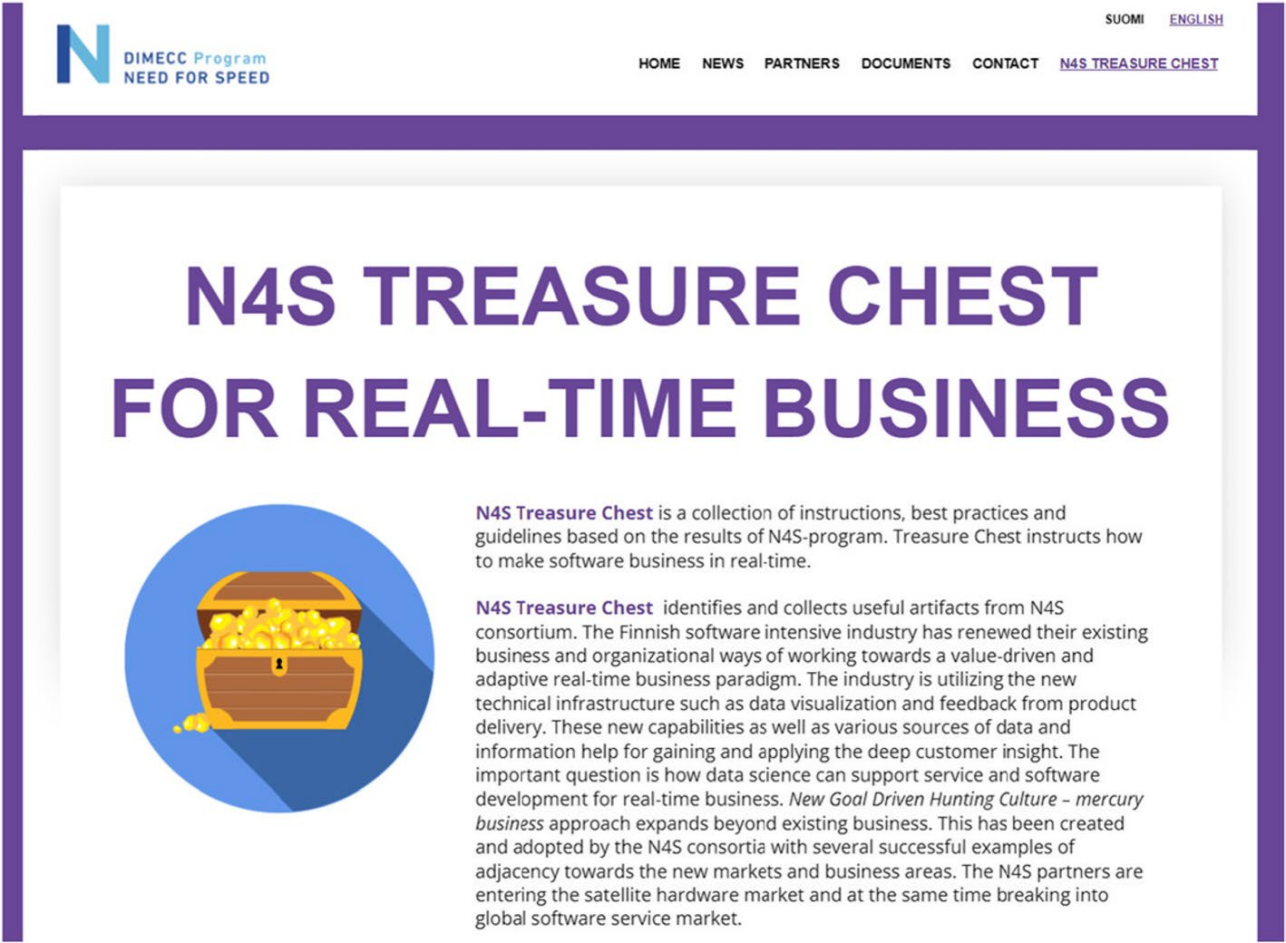
N4S Treasure Chest home page (excerpt)

The Treasure Chest was implemented as a publicly available web service. Figure 1 illustrates the web main page.

In the following, we first describe the organization, structure, and the information item categories of the Treasure Chest repository with illustrations of the actual web site. We then exhibit certain representative examples of each item type. Outstandingly, it contains, in addition to traditional research documents, narratives of the industrial learning experiences and more than 100 actionable knowledge items (called Gold Nuggets).

**Fig. 2:**
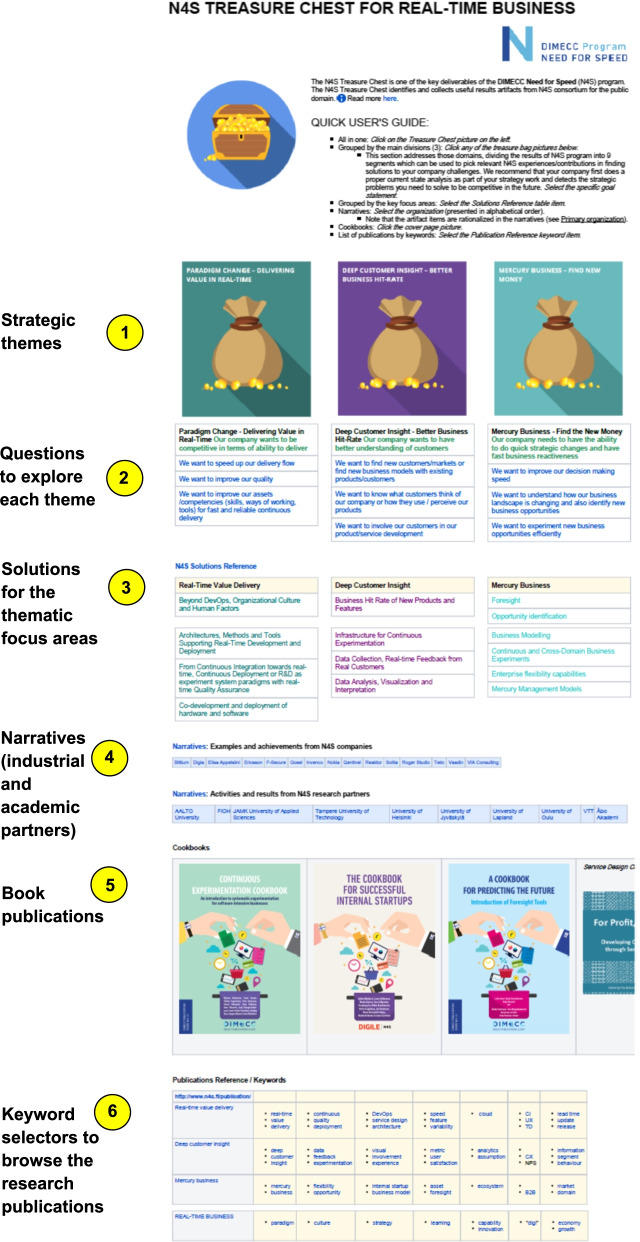
N4S Treasure Chest main organization and sectioning

The Treasure Chest consists of the following main parts and sections (see Fig. 2):

1. Main strategic themes
2. Guiding and triggering questions to explore each theme from typical angles
3. Solutions for the different research focus areas in each theme
4. Narratives from industrial and academic partners
5. Book publications
6. Keyword selectors (links) to explore the research publications

The six parts (1)–(6) marked in Fig. 2 work in practice for the user as follows:

1. By selecting (“clicking”) the icons of the three main themes, a list of all the related Gold Nugget knowledge items is displayed. The textual listing shows the titles of the items (in alphabetical order).
2. By selecting the different statements, designated subsets of the Gold Nuggets under the main theme are listed (in alphabetical order).
3. This section tabulates the research focus areas as stated in the N4S SRIA [18]. By selecting them, the corresponding subsets of the Gold Nuggets are listed (in alphabetical order).
4. Narratives are free-form reports of the N4S program achievements, works, results, and experiences written by each industrial and academic partner. Typically, they embed links to the related Gold Nuggets and research publications.
5. In addition to research publications, a collection of practitioner-oriented books were co-authored. This section provides links to access them.
6. During the program, more than 200 hundred publications (mostly research papers) were produced. Much emphasis, however, was also put in elaborating publications intended for practitioners by a professional journalist who was on program staff. This section of the Treasure Chest provides a tabularized set of keywords to browse them.

In the Treasure Chest, all the Gold Nuggets have the same defined format as presented in Table 2. The “Context” field intends to suggest where the particular Gold Nugget is most suitable to be applied. This is just an indicative suggestion as the real situations may vary. The two possible values are defined as follows:

- EXPLORATION: Discovering new product and service ideas and/or markets, inventing new business models; Feeding the realization for EXPLOITATION.
- EXPLOITATION: Implementing new products and services following the opportunities, developing new features for the products based on the feedback; Detecting potential new opportunities for further EXPLORATION.

**Table 2:** Gold Nugget template

<Gold nugget name>	
**Status**	*What is the status of the nugget?* *fixed options: Idea | Under development | Complete/Done*
**Attachments**	*Optional supplementary material of the nugget*
**Links**	**Relates**
	*Optional connections to related nuggets*
**Purpose**	*What is the purpose of the nugget, when to use it? Short summarizing description of the nugget.*
**Benefits**	*What benefits are expected from the use of the nugget?*
**Experiences and examples / cases**	*What kind of experiences are available? What business examples from partners are available?*
**Primary focus area**	*Fixed options based on N4S SRIA (Strategic Research and Innovation Agenda)*
**Additional information**	*Optional additional information of the nugget*
**Primary organization**	*Nugget “owner”*
**COP**	*Real-time value delivery | deep customer insight | mercury business*
**Context**	Explore | exploit
**Maturity of the organization**	Novice | practitioner | elite

In the “Maturity of the organization” field, the Novice-Practitioner-Elite ranking suggests the familiarity and experience of organization with respect to the Gold Nugget topic getting most benefits out of the nugget. The Novice-Practitioner-Elite ranking is, however, just an indicative suggestion as the real situations may vary.

Altogether, the Treasure Chest repository includes 171 Gold Nuggets. Table 3 illustrates one example. It was created collaboratively with research partners and an industrial company partner including a co-authored scientific conference paper.

**Table 3:** Gold Nugget example

CS/CX management dashboard		
**Status**	Done	
**Attachments**	<Illustrative handout> (pdf) <Research paper conference presentation> (pdf)	
**Links**	**Relates**	
	relates to	N4S continuous X capability development
	relates to	Framework for UX KPI dashboard
**Purpose**	Structure, analysis, and design of a B2B company CS/CX management system	
**Benefits**	Realizing systemic predictive B2B customer experience and satisfaction management: Customer satisfaction (CS) is continuously important in modern industrial business environments. However, it is inherently affective even in B2B contexts and thus not directly controllable. Satisfaction impacting customer experiences (CX), respectively, can be managed by the supplier company. The goals have to be made transparent to the entire organization for producing the experiences with their current status and projected progress. A transparent measurement system is thus needed.	
**Experiences and examples / cases**	<Poster> (pdf)	
**Primary focus area**	Data collection, real-time feedback from real customers Data analysis, visualization and interpretation	
**Additional information**	<Related program internal working items>	
**Primary organization**	University of Helsinki	
**COP**	Deep customer insight	
**Context**	Explore, exploit	
**Maturity of the organization**	Practitioner, elite	

The narratives (part 4 in Fig. 2) varied a lot for different industrial and academic partners reflecting the diversity and richness of the research, development, and innovation done during the N4S program. The following are some examples of the titles:

- *3 Years of continuous everything*
- *Amplifying the cycle between data and impact*
- *Continuous value definition (CE), actualization (CD), and determination (CX) practices and enabling capabilities development for real-time business*

**Fig. 3:**
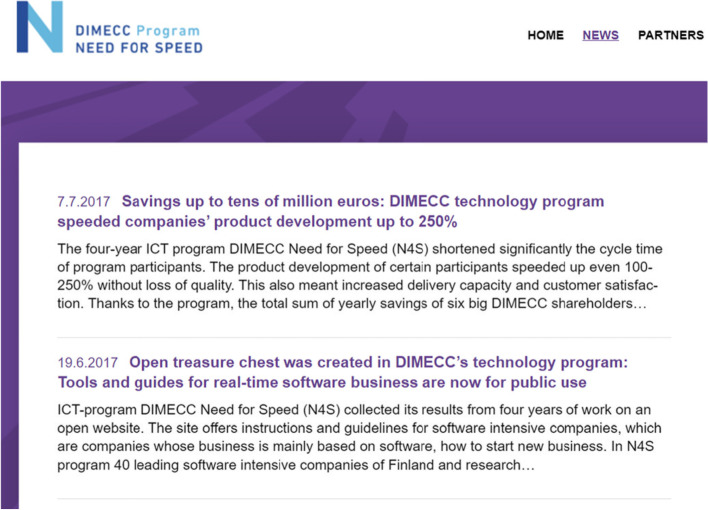
N4S Treasure Chest launching (excerpt)

**Fig. 4:**
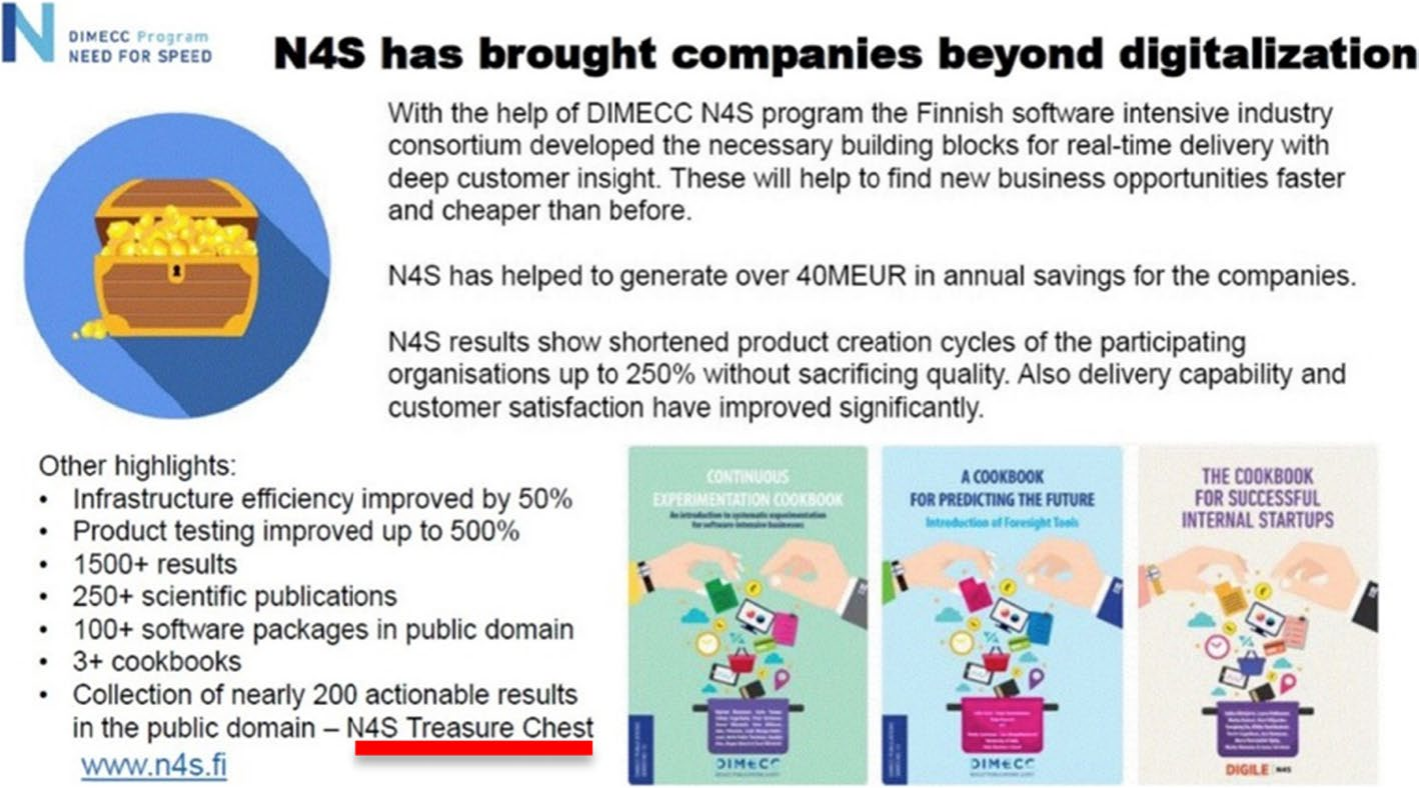
Treasure Chest in the N4S final outcomes reportage

Finally, the Treasure Chest launching was publicly promoted at the end of the N4S program in 2017 as shown in Fig. 3. In addition, the individual Gold Nuggets were advertised with a long series of Twitter messages by the end of 2017 (see @N4S_fi). The Treasure Chest was also one of the key outcomes highlighted in the N4S program final reporting as depicted in Fig. 4.

## Discussion

### Industry-academia cooperation in praxis

As the N4S program consortium comprised many industrial and academic partners (initially 25 and 10, respectively), there were many collaboration relationships and consequently various specific ways of working in cooperation. However, certain common patterns and features can be inferred. Table 4 decribes such. Here, we utilize a recent framework of evaluating IAC in Finland [20].

**Table 4:** N4S cooperation with respect to general IAC elements

IAC points [20]	Case N4S
*Who are the cooperating parties?* *What characteristics do they have?*	• Many of the N4S partners had been collaborating in a previous SHOK program (Cloud Software), so there were established relationships and even personal contact networks in place. • In addition, especially the quarterly joint review sessions provided face-to-face opportunities to make new contacts.
*Reasons and motives to start cooperating*	• SRIA: The distinct research goals for each strategic research theme (3) scoped and focused the overall research objectives. Each research partner was allowed to select the topics according to their research interests and expertise but in alignment with the industrial needs. • The industrial partners expressed and reasoned their goals and needs in the business case descriptions.
*What cooperation and how?* *Means of interaction, types, and outcomes*	• Joint publications writing • Workshopping (often in the company premises) • Quarterly reviews (e.g., joint presentations, demos, posters) • Common program information sharing system and repository (confluence) • Treasure chest: uniting collection and packaging
*What obstacles are there to start the cooperation or succeeding?*	• Who is and should be working with whom? • How much time and effort can each partner invest? • How can the academic partners gain appropriate and sufficient industrial domain knowledge?
*What factors enable successful cooperation?*	• Engaging shared efforts and targets (e.g., workshops, joint publications) • Personal contacts, trust, transparent and continuous information/knowledge sharing • Mutual flexibility and accommodating change in goal-setting and attainment

In hindsight, the SRIA envisaged a shared, energizing picture of the future. The three research themes depicted scenario paths to reach such futures. From the learning perspective, we can discern that the SRIA research goals and expected results actually defined intended learning outcomes (ILO) for everyone both in the industry and in the academia.

An important aspect of setting such an engaging vision was that the SRIA document was created with a joint effort by a large group (altogether 31 people, including the authors of the present paper) representing both the industrial partners and the research organizations (18 academic authors of which 9 professors). Interestingly, the actual writing was essentially accomplished in a couple of days during an intensive writing session at offsite premises. Before that, industrial needs and expectations were collected and captured as business case proposals and there were several collaborative preparation workshops (in 2013).

Notably, most of the industrial partner participants were in senior managerial positions in the companies. They had governing responsibilities and longer-term interests in developing the organizations also prior to and following the N4S program. Such setups can be seen to justify the relevance of the research goals and strengthen the industrial commitments. Furthermore, such resourcing is likely to encourage the academic partners to aim excellence in their research.

In the “Results” section, we have presented the Treasure Chest top-down and outside-in. However, in real life, we constructed it mostly the other way around during the 4-year program:

1. Publications (parts 5 and 6 in Fig. 2)
2. Gold Nuggets
3. Narratives (part 4 in Fig. 2)
4. Treasure Chest structure and compilation (parts 1–3 in Fig. 2)

Interestingly, the Treasure Chest was initially called just a “toolbox”. However, we realized that a more inspiring nickname would be beneficial, and the name “Treasure Chest” was jointly ideated. Following that, the knowledge items were coined as “Gold Nuggets”. (The English word “nugget” is easy to use also in oral Finnish.)

Overall, the motivation and interests for compiling and publishing the Treasure Chest grew and intensified gradually towards the end of the N4S program. There was a joint quest for collecting as many Gold Nuggets as feasible—even with some healthy competition.

There were certain jointly agreed rulings and policies underlying the creation and population of the Treasure Chest knowledge items. An overarching general idea was the quest to produce actionable knowledge for industrial use. It followed that purely theoretical research publications should be extended with application instructions in the Gold Nuggets (Attachments in Table 2). Another shared policy was that each academic research paper should have at least one industrial co-author.

We kept the threshold to submit new Gold Nuggets intentionally low. There were no formal acceptance gates. Instead, we relied on self-organization and self-assessment—and also healthy peer pressure. Basically, the only control rule was that everybody must adhere to the jointly agreed template (see Table 2). We firmly encouraged each partner to publish at least one Gold Nugget.

One way of measuring the industry-academia collaboration in practice is to quantify the number of Gold Nuggets created by different industrial and academic partners. For instance, in the focus area, “Beyond DevOps, Organizational Culture, and Human Factors” (see Fig. 2, part 3), there are altogether 40 items. Thirteen of them were submitted by industrial partners, 27 by research partners (universities and research institutes). Further quantification of the collaborations could be done by calculating, how many organizations (industrial and academic) were involved in creating each Gold Nugget.

Overall, an important part of the N4S industry-academia collaboration was the preparation of doctoral theses. Altogether, there are more than 15 theses done in conjunction to the program. 10 of them were defended during the program in 2014–2017. Markedly, there has also been a “long tail” since 5 theses have been defended after the formal program closing in 2019–2021, and some more are still expected to be completed. Usually, the thesis students (some of them industrial partner employees) worked closely together with industrial practitioners and academic researchers, and in many cases, the industrial companies were the subjects of empirical case studies. Notably, many of the research publications included in the article-based dissertations were parts of the Gold Nuggets.

In general, a principal motivation for all academic researchers is to be able produce new knowledge and publish it in high-quality scientific forums. This applies also in industry-academia collaboration settings. However, in such business-oriented environments industrial partners are typically geared towards shorter time horizons than is typically required by high-quality academic research work and expect readily applicable results for the current problems at hand.

In the N4S program case, that was not a significant problem, though. The relatively long time-span of the program (4 years, 2014–2017) was known in advance, and the resourcing was basically secured from the beginning, so the participating academic partners were able to concentrate on and commit to long-term research relationships with the industrial partners. Such settings are conducive for producing scientifically relevant journal articles, including longitudinal empirical studies. This is even fortified when the industrial participants are co-authors as was usually the case in the N4S.

### Comparing and contrasting

The first Finnish SHOK programs were started in 2007. Related to the N4S case, a predecessor was the Cloud Software (CSW) program in 2010–2013. The authors were participants also in that SHOK program.

During the active years, the SHOK program system has been evaluated externally [21, 22]. The assessments reported both benefits and challenges with constructive criticism. The assessments concentrated on the SHOK level (TIVIT/DIGILE in our case) rather than individual programs like N4S. In Table 5, we present general key challenges indicated in those SHOK evaluations and mirror the N4S program case against them.

**Table 5:** N4S with respect to general SHOK challenges

SHOK evaluation issues (prior to N4S) [22]	Case N4S
*SRAs too all-encompassing, multiple (too many), and some internally contradictory objectives*	
Sharp agenda focus	• SRIA: Well-defined focus areas (4+4+6) under distinct but logically interrelated research themes (3) • Treasure chest: Categorization according to the focus areas (c.f., Fig. 2, part 3)
*Tensions between the short-terms industrial interests (incremental innovation) and the longer term perspective of high-quality, impactful (even breakthrough) scientific research*	
Industrial renewal, transformational research: lack of innovative results, novel and path-breaking research outside; too consensus-oriented	• SRIA: The stated breakthrough targets aimed to “create the foundation for the Finnish software intensive businesses in the new digital economy” so that the “Finnish software-intensive industry has renewed their existing business and organizations”. • The aim was to “act as a forerunner in catalyzing systemic transformations in different industries”. • However, during the program we did not much “see the global digital services business growing in Finland and completely new Finnish brands in digital business introduced”.
Fully engaging academics: scientific research in relatively small roles, lack of internationalization and global dimensions	• SRIA: Created jointly by the academic and industrial partners. The academic researchers were “equal partners” and respected stakeholders. • The scientific research ambition was high with stretched, even world-class aims. There were plenty of scientific research opportunities for each partner to contribute with high-quality research. • The internationalization and global collaboration were limited and not emphasized although in the beginning there were certain engaging connections.
Strategic alignment (industry and academia): lack of cross-disciplinary and sector-transgressing themes, stretching beyond sectoral boundaries	• SRIA: The very premise was to advance beyond digitalization. • The research themes were principally domain- and sector-independent. Digitalization capabilities are by nature relevant in and across all industries. The “long-term plan of N4S is to serve other companies where software plays a dominant role”.
*Lack of consistent performance measurements and systematic monitoring (KPIs)*	
Transparent interactive progress monitoring	• SRIA: Key goals and measurements related to the breakthrough targets • Dashboards (JIRA) • Quarterly joint reviews (presentations, posters, demos), with “best paper” recognitions • Treasure Chest: # of the Gold Nuggets

The overall N4S collaborative RTDI strategy and program management approach has been explained and evaluated in previous publications [10, 11]. The present paper adds on them by presenting and scrutinizing the Treasure Chest and its creation process in that context.

In Table 1, we have summarized typical challenges and success factors in IAC research relationships. With the Gold Nuggets, the academic research efforts and contributions were naturally oriented towards applicable and relevant industrial needs linking to the daily work of the industrial partners since the industry-driven program target was to produce actionable knowledge for distinct purposes. That helped ensuring the practicality and applicability while not overly constraining the academic research. The Treasure Chest facilitated natural close collaboration and was a shared vehicle for being agile toward industry needs and organizing and executing the joint work in agile ways.

Many related investigations on IAC have suggested good practices and recommendations. For instance, it has been recommended to facilitate results that have deployment impact since targeting immediate goals related to current industry needs are more likely to succeed keeping research projects well aligned—while still allowing innovations to emerge [13]. Those were very much the intentions of the Gold Nuggets. We could also see that the Treasure Chest and the Gold Nugget creation process brought mutually beneficial continuous collaboration ceremonies thriving accomplishments together [14].

In general, we posit that the forward-looking approach set in the SRIA amplified the role of futures knowledge and futures consciousness both in the research organizations and in the industrial companies [23]. This could be seen in the titles of the narratives (part 4 in Fig. 2), such as:

- *The need for speed increases all the time*
- *The N4S as a growth enabler*
- *Growth and diversification in ever faster paced markets*
- *Towards real-time business*

The Finnish government has emphasized building of internationally attractive knowledge clusters, networks, and innovation systems with leveraging the skills in higher education institutions to accelerate R&D&I for supporting and revitalizing businesses by 2030 [24]. We maintain that the N4S IAC has advanced such aims.

Overall, from our local self-evaluation perspective and based on our participatory experiences, the N4S program was perceived to be by and large successful. Table 6 contrasts actual outcomes with the original targets stated in the SRIA.

**Table 6:** N4S goals and measurements related to the breakthrough targets

Goals and metrics [18]	Actual results (see Fig. 4)
REAL-TIME VALUE DELIVERY: *Lead time decreases 20% annually.* • *Lead-time decreases annually towards right-time, real-time value delivery targets.* • *Quality of products or services increases (e.g., less bugs or better customer satisfaction).*	• Shortened product creation cycles up to 250% without sacrificing quality • Delivery capability and customer satisfaction improved significantly. • Infrastructure efficiency improved by 50%. • Product testing improved by 500%.
DEEP CUSTOMER INSIGHT: *Active user base of affected services is doubled by 2017.* • *Business opportunities identified and systematically considered/analyzed*	• Possibly not achieved (insufficient evidence)
MERCURY BUSINESS: *Revenue growth from 10% annually to >50% by 2019* • *New opportunities identified and utilized* • *New products and services outside existing business* • *Start-ups emerged*	• Possibly not achieved (insufficient evidence)
*Finnish digital businesses have entered into entirely new markets, customer segments, and several completely new brands are introduced.*	• Not conceivable
*Finland will be a new innovation and investment hotspot.*	• Not conceivable

The actual results concentrate on the Real-Time Value Delivery research theme. This is also visible in the Treasure Chest in the sense that the number of the Gold Nuggets is largest in that category (c.f., Fig. 2, part 1). This is understandable and justified on the grounds that this theme was closest to daily industrial operational practices, and many companies had been working on such developments already prior to the N4S program. Moreover, it creates foundations for the new business capabilities (Mercury Business).

Admittedly, as shown, the planned targets were only partially achieved. However, the intention of the original targets was to be extremely ambitious and even partial achievement may be considered as success. Furthermore, as noted in the external evaluations, many of such performance targets are not straightforward to measure and the overall picture remains partially fuzzy due to the lack of comparative data—perhaps even impossible to assess [22, 25].

In addition to the metrics and targets presented in Table 6, the SRIA stated as an expected outcome that the “results will be packaged as capabilities that can be disseminated and exploited widely in the Finnish economy to enhance global competitiveness that will attract foreign investments in Finland” [18]. Markedly, the Treasure Chest fulfils exactly that.

Markedly, although the SHOK programs have been criticized and many challenging issues have been noted as discussed above, the assessments have also recognized many positive effects and impacts [17, 21, 22]. One of the main positive aspects has been acknowledged to be the industry-driven large-scale collaboration and with new partners for more ambitious, open, and committed networked research. It has created networks and collaborations between firms, universities, and research institutes that would probably not have been possible otherwise. In our view, that was also one of the principal positive factors in the N4S program.

The Finnish SHOK system was discontinued in 2016 [17]. We are not in a position to judge, why that governmental decision was made and on which grounds. It was related to the restructuring of the Finnish research and innovation funding system.

The general long-term trends of RD&I investments and industry-academia collaboration in Finland have been downwards in 2010–2020 [20, 26]. Both the public funding and the academic research funding received from industry have been decreasing. In addition, industry-academia collaboration as measured in terms of the number of joint publications has been declining.

Naturally, such overall governmental and industrial factors may have influenced the decisions to discontinue the Finnish SHOK system. However, no clearly better new supporting systems have been created since then either [20]. That has likely had negative consequences for long-term research and achieving the original ambition goals of the SHOK system stated in 2005 in the expected 5–10-year time horizon.

### Implications

#### Lessons learned

During the entire N4S program period in 2014–2017, we attempted to continuously learn and accordingly improve our I-A collaboration ways of working and practices. Moreover, we sustained several beneficial practices throughout the program. We can now draw several managerial implications from these lessons learned.

Especially the following ones we advocate to adopt, foster and sustain:

- Guiding and energizing shared vision from the beginning (picture of the future)
- Coaching and uniting leadership—respected by both the academic and industrial partners
- Agile ways of working and mindset (e.g., quarterly joint review meetings)
- Supporting ICT and communications infrastructures, competences, and resources—considering also the life after the formal closure of the collaboration program (e.g., preserving the repositories and web pages for future access and use; In our N4S case, we managed to secure the technical system availability including hosting the Treasure Chest for a couple of years.)

In addition, the following principles could in our experiences be worth trying and nurturing:

- Consciously alternating between exploration (problem definition) and exploitation (problem-solving).
- Balancing between theory (e.g., conceptual models) and practice (actionable knowledge)

In contrast, there are also many things to avoid. One of the most critical ones experienced during the our N4S case were external mid-course funding cuts. While such external factors may in practice be beyond the direct control of the I-A collaboration, their detrimental effects for instance on the team spirit should be taken into account and alleviated as much as possible.

Remarkably, even though the actual research program was formally closed in 2017, the IAC networks formed during the program have continued informally—as for instance the co-authoring of this paper exhibits. We have found such resulting networks and connections highly valuable both for academic and industrial organizations and persons alike. In fact, what could be perceived as the “N4S spirit” appears to stay alive (c.f., Twitter: @N4S_fi).

A general, principal problem in IAC research programs tends to be the basically different and sometimes even conflicting motives and incentives of industrial and academic participants. While industrial partners are inclined to solve their immediate practical problems at hand, scholarly academic researchers want to discern the fundamental questions, understand the underlying reasons, and produce new scientific knowledge to publish in academic forums (preferably top journals).

In the N4S program, the SRIA was jointly created by the key academic and industrial participants so that both parties could have their interests incorporated. Many of the large, incumbent industrial partners had been initiated for instance agile/lean improvements and even transformations already prior to the N4S program, so they were also inclined to longer-term research-oriented efforts. Design science was recognized in the SRIA as a viable research method. Overall, the stated strategic research themes (3) with the defined focus areas were ambitious, even world-class goals for high-quality academic research while at the same addressing such areas and questions which served the industrial needs, so the setting allowed “win-win” relationships.

In addition to the SRIA, the jointly created Treasure Chest helped aligning and converging academic and industrial aims. The Gold Nuggets were expected to provide actionable knowledge. Consequently, they required both producing knowledge (i.e., scientific research) and making it exploitable for practical industrial use. Many Gold Nuggets included co-authored empirical case study publications.

A survey study among Finnish doctorates who have exited academic investigated two-way knowledge transfer (information and knowhow) between universities and the “surrounding society” including industry with a dilemma approach [27]. The prevailing basic dilemma related to the third mission of universities is in doing “pure” scientific research vs. making knowledge applicable. The study suggested three strategies for bridging such gaps: universities’ building stronger stakeholder relationships, knowledge co-production with and by the stakeholders considering the usability of the research results, and enforcing science communication with related incentives. Our N4S experiences and results suggest that the SRIA and the Treasure Chest were primes towards such stronger stakeholder relationships and co-production. The jointly created Treasure Chest was a concrete artefact for publicly communicating the developed knowledge in applicable forms.

#### Reflections on learning and futures research

We are now in a fitting position to reflect the N4S program IAC and the Treasure Chest from learning and futures research perspectives in more general. This brings up certain theoretical implications.

Considering futures research, the key forward-looking elements were the picture(s) of the futures and their associated scenario paths. In the beginning, the SRIA stated the following grand vision [18]:

- “By 2017, the Finnish software intensive industry is the recognized leader in business innovation and fast implementation of products and services in the digital economy”.

Moreover, it designated the following overall scenario path to reach that desired picture of the future:

- “This has been achieved by adopting a real-time experimental business paradigm, providing instant value delivery based upon deep customer insight”.

Following that line of heading, the related research focus areas, concrete goals, and provisionally envisioned results outlined in the SRIA oriented and aligned energetically and enthusiastically the collaborative research, development, and innovation activities right from the beginning.

Markedly, foresight was an intrinsic aspect of the N4S three strategic research themes which create together a real-time business system. Especially, the Deep Customer Insight theme aimed to create advanced capabilities for continuous environment scanning and recognizing new and changing customer/user behaviors and needs. The Mercury Business theme then intended to flexibly and continuously prospect new businesses following the customer insights. The Real-Time Value Delivery theme targeted to bring up high-performing product and service creation capabilities (both technical and organizational) so that the business opportunities can be sized in a continuous yet sustainable fashion. Foresight was explicitly one of the focus areas for the Mercury Business (c.f., Fig. 2, part 3), and one of the program outputs was a basic guidebook (“cookbook”) for practical foresight activities (c.f., Fig. 4). However, of all the SRIA focus areas the Foresight contained the smallest number of Gold Nuggets in the Treasure Chest (7).

In principle, all the over 100 Gold Nuggets in the N4S Treasure Chest can be considered as weak signals in the same manner as for instance the 100 opportunities identified and evaluated in the Radical Technology Inquirer for anticipation of technological breakthroughs [28]. The Gold Nuggets were—at the time of their discovery and packaging (2017)—considered preferable and prospective for new needs and opportunities. Consequently, they can nowadays (at the time of this writing after 5 years in 2022) be re-evaluated with respect to seeing them as weak signals. Some of them may have remained such, but some may have become current realities and even mainstream. Furthermore, some may include still open research problems and questions serving as inputs for future research agendas. Thus, for example the Gold Nugget illustrated in Table 3 can be evaluated with respect to its (customer satisfaction/experience management) relevance and importance in contemporary and future business environments. It is reasonable to judge now that it has not been outdated—on the contrary, perhaps being even more important today in increasingly customer-centric business models.

Considering learning, the narratives included in the Treasure Chest (part 4 in Fig. 2) can be seen as learning reports. For example, the narrative of one industrial partner illustrated the company development path towards real-time business and way of working started already well before the N4S program. That is, the N4S program activities and developments were reflected in the larger context and longer-term time scale. The narrative then shows applications of different N4S research areas at the company during the program with the achieved advancements and improvements.

Finally, the SRIA was also expected to influence curricula development. Especially, the aim was to contribute to the integration of different disciplines. Multidisciplinary collaboration was intended to raise to higher levels. Considering current levels and extends of cross-cutting digitalization, such multidisciplinary curricula can be seen ever more necessary. Notably, altogether the three N4S research themes required competences from multiple disciplines ranging from core software engineering and production to software-oriented business competences. A question remains, which higher education institutes (in Finland) offer such varieties and combination possibilities of study tracks and courses in their curricula.

#### Future work

In the N4S, the Treasure Chest was essentially created and compiled during the final phase of the program (2016–2017). Consequently, it left room for further development and evaluation. The following ones are such immediate opportunities:

- How extensive and intensive was the collaboration (e.g., network density [4])? This could be illuminated for instance by quantifying, how many co-authors from different industrial and academic organizations there were in the different publications (around 200, see https://n4s.dimecc.com/en/documents/articles/). In a similar fashion, the Gold Nuggets could be quantified with respect to with which academic partners each industrial partner collaborated in creating them.
- By performing a cross-referencing analysis, it would be possible understand how the different Gold Nuggets are interrelated. Similarly, it is possible to cross-analyze the different narratives (26, see part 4 in Fig. 2) for discovering potential interrelationships and synergies. For example, the word *continuous* appears in 5 titles and the word *value* in 3 titles.
- Assessing the reuse potential of the Treasure Chest and the individual Gold Nuggets
- Utilizing the Treasure Chest for software engineering educational and future IAC research purposes

**Fig. 5:**
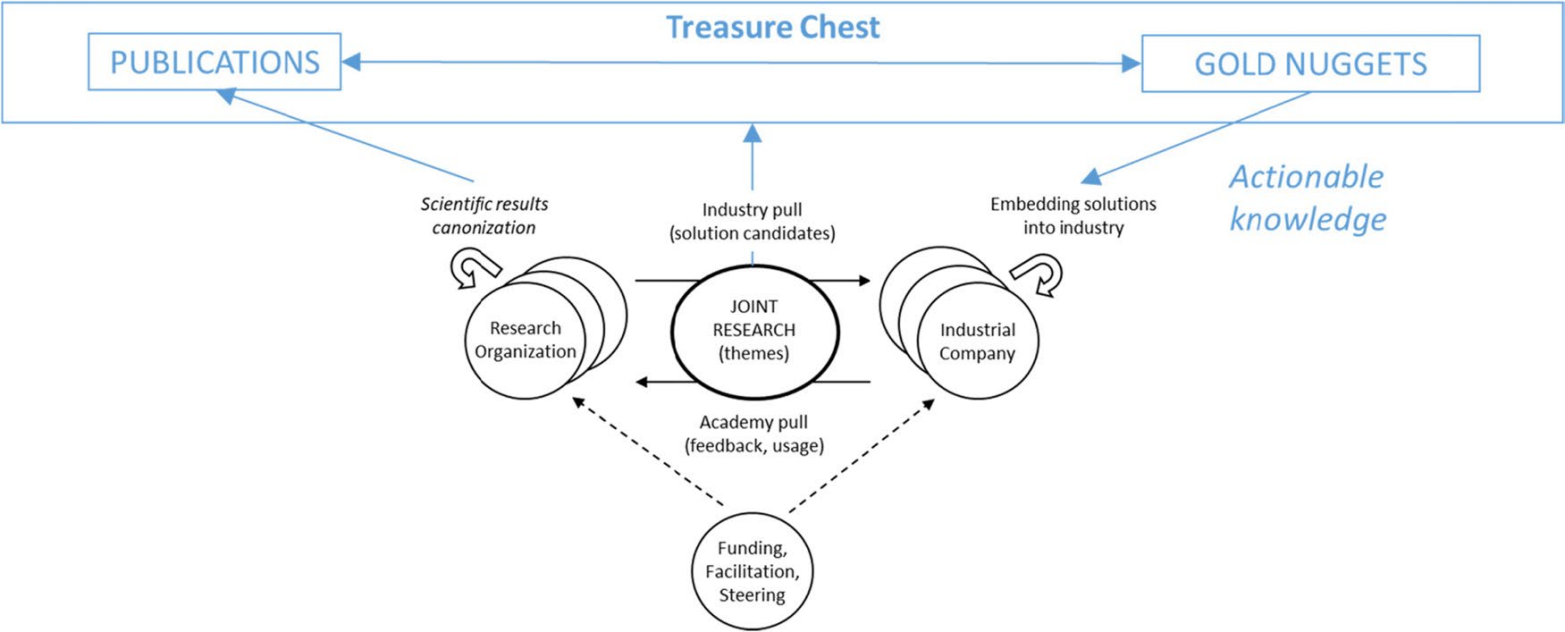
Extended technology transfer model for multi-party IAC research (derived from [11])

Furthermore, more theoretical advances could be pursued following the previous suggestions on agile IAC and software technology transfer approaches [10, 11]. Figure 5 proposes an enhancement of our technology transfer model coupled with the Treasure Chest.

Modern software researchers should exercise even continuous foresight activities especially by scanning the current and also emerging software production and application environments. Currently for instance the EU research agendas are geared towards digitalization and green transitions. Consequently, a future strategic software research agenda quest could be a combination of digital transformations and green sustainability transitions. Notably, many advanced (smart) systems for example in manufacturing environments are increasingly software-intensive. Interestingly enough, the general idea of the N4S program was to advance beyond digitalization. A future “N4S 2.0” SRIA could thereby be envisioned. For instance, the Futures Map approach could potentially be utilized in doing that [29].

## Conclusions

In this paper, we have investigated a recent (2014–2017) large-scale IAC program case (Need for Speed, N4S) from learning and futures research perspectives. We have reviewed, what the program had done and achieved, and evaluated the results of the study. Especially, we have presented a collaboratively created and publicly available shared digital knowledge repository called Treasure Chest produced during our case program. The starting point was a jointly created Strategic Research and Innovation Agenda (SRIA), which defined the main research themes with intended focus areas and listed motivating and orienting research goals and questions to begin with.

The Treasure Chest was ideated and compiled during the last phase of the 4-year program. However, already in the beginning of the program, the SRIA encouraged towards such by stating that the results will be packaged and disseminated for wide exploitation.

In the future, large-scale IAC projects should be more and more commonplace since major innovations are hardly ever done in isolation, not even by the largest companies. Moreover, most current grand research and development challenges require multidisciplinary cooperation and especially software systems are ever more connected and cross-cutting. Both intra-organizational and inter-organizational co-creation and learning networks are increasingly critical success factors. Collaborative learning capabilities will thus be required more often from all the participating parties.

The N4S SRIA defined the main research themes, focus areas and goals with orienting, triggering research questions to begin with. Moreover, it envisioned the desired picture of the future for the entire program consortia. In conclusion, our suggested vision is that such transparently shared, rich outcome goals with continuous integrative collection of the results are keys to effective networked I-A learning in collaborative R&D&I journeys. In the N4S case, the collectively produced Treasure Chest was the concrete manifestation of the successful IAC and its joint learning outcomes at the end of the program and even a couple years after.

Finally, we maintain that the principal research problems stated in the N4S SRIA in 2015 are still (in 2022) very much valid. Interestingly enough, digitalization has significantly accelerated in many fields during the past year of the COVID-19 pandemic. Moreover, the current EU aims for supporting digital and green developments are topical for most every industrial sector for several years to come.

It is perhaps fair to say that the grand vision of the program was not fully was achieved by 2017. However, considering futures research, that is the very idea of a visionary picture of the future as an ideal “dream” state. We maintain that the vision was—and still is—desirable and plausible, and the N4S IAC program progressed significantly in the scenario path towards that vision. During the 4-year journey, we learned a lot together as manifested by the Treasure Chest.

Overall, a key success factor of the industry-driven N4S program was that it created and sustained an environment and atmosphere, which was conducive for mutually beneficial and energizing long-term (4 years) industry-academia collaboration. The jointly created, future-oriented SRIA chartered highly ambitious research goals suitable and attractive for all the academic partners and researchers to contribute on the one hand and the designated focus areas and goals were relevant and rational for the industrial partners on the other hand. With such headings and settings, collaborative participatory research was supported and lucrative.

## Data Availability

There are no supplementary data sources.
